# Open versus minimally invasive percutaneous surgery for surgical treatment of thoracolumbar spine fractures- a multicenter randomized controlled trial: study protocol

**DOI:** 10.1186/s12891-019-2763-1

**Published:** 2019-08-31

**Authors:** Helton L. A. Defino, Herton R. T. Costa, Altacílio A. Nunes, Marcello Nogueira Barbosa, Valéria Romero

**Affiliations:** 1 Faculty of Medicine, University of Sao Paulo, Ribeirao Preto, Brazil; 2 Department of Medical Clinic, Faculty of Medicine, University of Campinas, Campinas, Brazil

**Keywords:** Spine, Spinal injuries, Fractures, Bone, Mini-open surgery, Pedicle screw

## Abstract

**Background:**

Thoracolumbar fractures are most frequent along the spine, and surgical treatment is indicated for unstable fractures. Percutaneous minimally invasive surgery was introduced to reduce the pain associated with the open posterior approach and reduce the morbidity of the procedure by avoiding damage and dissection of the paravertebral muscles. The goal of this study is to compare the surgical treatment of fractures of the thoracolumbar spine treated by the conventional open approach and the percutaneous minimally invasive approach using similar types of pedicle spine fixation systems.

**Methods/designs:**

This study is designed as a multi-center, randomized controlled trial of patients aged 18–65 years who are scheduled to undergo surgical posterior fixation. Treatment by the conventional open approach or percutaneous minimally invasive approach will be randomly assigned. The primary outcome measure is postoperative pain, which will be measured using the visual analogue scale (VAS). The secondary outcome parameters are intraoperative bleeding, postoperative drainage, surgery time, length of hospital stay, SF-36, EQ-5D-5 l, HADS, pain medication, deambulation after surgery, intraoperative fluoroscopy time, vertebral segment kyphosis, fracture vertebral body height, compression of the vertebral canal, accuracy of the pedicle screws, and breakage or release of the implants. Patient will be followed up for 1, 2, 3, 6, 12 and 24 months postoperatively and evaluated according to the outcomes using clinical and radiological examinations, plain radiographs and computed tomografy (CT).

**Discussion:**

Surgical treatment of thoracolumbar fractures by the open or percutaneous minimally invasive approach will be compared in a multicenter randomized study using similar types of fixation systems, and the results will be evaluated according to clinical and radiological parameters at 1, 2, 3, 6, 12 and 24 months of follow-up.

**Trial registration:**

ClinicalTrial.gov approval number: 1.933.631, code: NCT03316703 in may 2017.

## Background

Thoracolumbar spine fractures are the most frequent fractures of the axial skeleton, and approximately two-thirds of these fractures occur between T11 and L2 [1]. Surgical treatment has been indicated for unstable fractures to stabilize the injured vertebral segment and allow early rehabilitation without ortheses or bed rest [2]. Conventional surgical treatment is performed by posterior fixation and arthrodesis of the injured vertebral segment using a pedicle fixation system with or without arthrodesis. This approach is performed via the open posterior approach over the midline [3–5].

Less invasive procedures are a growing trend in spinal surgery with an aim of minimizing tissue trauma, reducing blood loss and facilitating patient recovery. The use of percutaneous pedicle screws is part of minimally invasive procedures of the spine, and they can be applied in surgical procedures that use a fixation system mainly for treatment of fractures, degenerative diseases and tumors [6, 7].

The use of percutaneous pedicle screw fixation for the treatment of thoracolumbar fractures has been reported and is believed to have the advantages of the open approach (i.e., restoration of the alignment and stabilization of the vertebral segment) without its morbidity. Minimally invasive percutaneous pedicle screw fixation obtains results similar to those of traditional open surgery and has the advantages of less trauma, bleeding, and pain and rapid post-operative recovery [8, 9].

Recently, reports of the outcomes of surgical treatment of thoracolumbar spine fractures through the percutaneous pedicle spine have been increasing. However, randomized clinical trials are still rare, and more of these trials are needed to demonstrate the advantages of minimally invasive percutaneous pedicle screw fixation over the traditional open technique. The objective of the study is to compare the surgical treatment of fractures of the thoracolumbar spine using the conventional open approach or the minimally invasive percutaneous approach for stabilization of the affected vertebral segment with a similar type of pedicle spinal fixation system.

## Methods/design

This will be a multi-center randomized controlled trial to assess the effect of minimally invasive percutaneous surgery versus conventional open surgery in patients with thoracolumbar spine fractures with an indication for posterior stabilization. The primary endpoint will be the postoperative pain, evaluated through the visual analogue scale (VAS) and compared between the patients subjected to minimally invasive percutaneous surgery and those subjected to conventional open surgery. The follow-up period will be two years, during which time the patients will fill out questionnaires and be evaluated both clinically and radiologically. Conventional open surgery and minimally invasive percutaneous surgery will be compared in patients with thoracolumbar spine fractures with indication for posterior stabilization. The study is designed as a multi-center (five centers) study. The follow-up period is 2 years, during which time the patients will fill out questionnaires and be evaluated both clinically and radiologically.

### Patient selection

Patients of both sexes aged 18–65 years with fractures of the thoracolumbar spine and an indication for posterior fixation (one-level fracture, regional kyphosis > 30 degrees, regional kyphosis with osseous or ligamentous lesion, collapsed vertebral body > 50%, three vertebral column lesion, or unstable lesion) will be enrolled.

The indication and options for surgical treatment will be presented to patients who meet the inclusion criteria (Table 1). The patient will be informed about the objectives, investigative design, assessment and possible advantages and disadvantages of the proposed treatment. The patient will be asked to sign the informed consent form. Pre-operative baseline data will be collected for the outcome scores, patient demographic characteristics and classification and characterization of the injured vertebral segment.

**Table 1:** Selection criteria

Inclusion criteria	
One level fracture	
Fracture T11-L5	
Adult (18–65 years old)	
Indication for posterior fixation	
Exclusion criteria	
More than one level fracture	
Osteoporotic fracture	
Pathological fracture	
Previous spinal surgery	
Spinal deformity	
Mental illness	
TCE	
Indication for posterior canal decompression	

Neurological deficit is considered in the evaluation but not in the inclusion or exclusion criteria, unless there is an indication for posterior decompression. The patients with neurological deficit that do not need a posterior decompression can be enrolled in the study if there is an indication for posterior fixation. In patients with total neurological déficit and no indication for canal decompression the treatment can be performed just by a posterior approach. In patients with neurological deficit with indication for anterior decompression of the vertebral canal after posterior fixation can also be included in the study.

### Informed consent and patient safety

The patient will be asked to sign the informed consent form. Pre-operative baseline data will be collected for the outcome scores, patient demographic characteristics and classification and characterization of the injured vertebral segment. A patient who meets the inclusion criteria will be informed about the study. The therapeutic options as well as their benefits and risks will be explained, and the patient will be free to participate in the study. If the patient is willing to participate in the study, written informed consent will be obtained.

The patients will be notified that they are free to withdraw from the study at any time. Adverse effects or complications will be treated and followed up until the issue is stable or resolved. The study was submitted to and approved by an institutional review board (IRB) trial registration, ClinicalTrials.gov Protocol Registration and Results System (PRS): NCT03316703, Approval Number: 1.933.631, study start in june 1, 2017.

### Randomization

Patients who meet the inclusion criteria and have provided informed consent will have their fractures classified according to the system proposed by Magerl et al. The randomization process will be performed for each fracture type (A, B and C). The patients will be randomly allocated into the following two groups: A- conventional open surgery and B- minimally invasive percutaneous surgery. The randomization will be performed via a centralized system. The research team will contact the central randomization system informing the type of fracture (A, B or C). The type of treatment will then be assigned by this system, with the patient being subsequently allocated to the appropriate group. We will use envelopes for each fracture type, following the CONSORT (Consolidated Standards of Reporting Trials) guidelines. The randomization will be performed using numbers generated by a specific software [MedCalc Statistical Software version 18.11.3] and will obey the 1:1 ratio for each group [MedCalc Software bvba, Ostend, Belgium; https://www.medcalc.org; 2019].”general anesthesia. Stability of the spine will be achieved by bilateral short-segment pedicle instrumentation. Pedicle screws will be inserted into the vertebral body one level above and below the fractured vertebra. The fractured vertebra will also be included in the fixation. In the fractured vertebra, a short pedicle screw will be inserted uni- or bilaterally according to the degree of pedicle involvement.

The spine fixation system is composed of a 6.5-mm-diameter polyaxial pedicle screw attached to 5.5 mm rods. The vertebral fixation system will be applied through the conventional open approach in group A (open approach) and percutaneously without open exposition of the injured vertebral segment in group B (minimally invasive percutaneous surgery). Posterior fusion using bone grafts or bone substitutes will not be performed in either group. External immobilization will not be used after surgery, and the patients will be allowed to walk according to their general clinical picture and pain.

### Outcome measurements

At admission, demographic, clinical and radiographic data for the patients will be obtained for characterization and classification of the fracture. After randomization, allocation of the patient into the study group and definition of the type of treatment to be performed, parameters related to the preoperative period will be acquired (Table 2).

**Table 2:** Variables collected in the preoperative period

Level of fracture	
Neurological status (ASIA scale)	
Type of fracture (Magerl’s classification)	
Comorbidities	
Associated lesions	
Fracture of upper limbs	
Fracture of pelvis or lower limbs	
Charlson comorbidity index	
EUROQL-5	
Short-form SF-36	
HADS (Hospital Anxiety Depression Scale)	
Pain (VAS)	
AP Rx- vertebral alignment	
Lateral Rx- height of the fractured vertebral body, height of the vertebral body above and below the fractured vertebra, kyphosis of the injured vertebral segment, kyphosis of the fractured vertebra, sagittal index (Farcy index)	
CT- compression of the spinal canal, sagittal and coronal diameters of the spinal canal, lamina fracture	

Intra-operative parameters related to the surgical procedure will also be acquired (Table 3).

**Table 3:** Variables collected in the intra-operative period, related to the surgical procedure

Type of approach (open/percutaneous)	
Operating time	
Intraoperative blood loss	
C-arm exposure	
Surgical drain	
Implants – mono or polyaxial screw/diameter/length/rods	
Intra-operative adverse events – (anesthesia, hemorrhage, dural lesion, neurological lesion)	
Intra-operative change of implants	

During the immediate postoperative period up to the moment of hospital discharge, data regarding the clinical evolution of the immediate postoperative period, use of analgesic medication, immediate postoperative complications, and postoperative imaging tests will be collected to evaluate the radiographic parameters (Table 4).

**Table 4:** Variables collected in post-operative and hospital discharge parameter

Drainage – (48 h)	
VAS – 3 days postoperative	
Deambulation	
Complications – superficial infection, deep infection, neurological lesion, vascular lesion, hematoma	
Analgesics- drug, dosage, period of administration	
Antibiotics- drug, dosage, period of administration	
Number of the days from surgery till hospital discharge	
Adverse effects	
Reoperation	
AP Rx- vertebral alignment	
Lateral Rx- height of the fractured vertebral body, height of the vertebral body above and below the fractured vertebra, kyphosis of the injured vertebral segment, kyphosis of the fractured vertebra, sagittal index (Farcy index	
CT- compression of the spinal canal, sagittal and coronal diameters of the spinal canal, position of the screws inside the pedicle	

After hospital discharge, the patients will have follow-up visits at 1, 2, 3, 6, 12 and 24 months after surgery for clinical and radiological evaluations (Table 5).

**Table 5:** Variables collected and to be evaluated during follow-up (1, 2, 3, 6, 12 and 24 months)

Short-form SF-36	
HADS	
Dennis’s pain scale	
Dennis’s work scale	
EUROQOL-5	
VAS	
Roland Morris Disability Questionnaire	
Complications	
Analgesics- drug, dosage, period of administration	
Adverse effects	
AP Rx- vertebral alignment, implant breakage, loosening	
Lateral Rx- height of the fractured vertebral body, height of the vertebral body above and below the fractured vertebra, kyphosis of the injured vertebral segment, kyphosis of the fractured vertebra, sagittal index (Farcy index), implant breakage, loosening	

### Primary outcome measure

The pain intensity after the surgical procedure will be evaluated using the VAS. The pain is related by the patient on a 100 mm horizontal visual analog scale. The two ends of the scale are 0 mm (no pain) and 100 mm (the most terrible pain that I can imagine). The patient will be asked to mark the scale based on their average pain during the evaluation period. The pain intensity will be evaluated before and after the surgical procedure. After surgery, pain will be evaluated on the first three postoperative days and at 1, 2, 3, 6, 12 and 24 months after the surgical procedure. Pain will also be evaluated according to Dennis’s scale at the 1, 2, 3, 6, 12 and 24 month follow-up visits.

### Secondary outcomes

The EUROQOL-5 and Short-form health survey (SF-36) will be used as generic quality of life questionnaires. The Roland Morris disability questionnaire will be used as a specific outcome for low back pain, and the capacity to work will be evaluated according to Dennis’s work scale. The HADS (Hospital Anxiety Depression Scale) will be evaluated preoperatively and during follow-up.

### Radiographic images

The fracture will be evaluated on plain radiographs (AP and lateral) and CT preoperatively and after the surgical procedure. During follow-up (1, 2, 3, 6, 12 and 24 months), only plain radiographs (AP and lateral) will be used for evaluation.

On the plain radiographs, the AP alignment and deviation will be evaluated. On the lateral radiographs, the height of the fractured vertebral body, height of the vertebral body above and below the fractured vertebra, kyphosis of the injured vertebral segment, kyphosis of the fractured vertebra, implant breakage or loosening will be evaluated. On CT, the compression of the spinal canal, sagittal and coronal diameters of the spinal canal, lamina fracture and position of the screw inside the pedicle will be evaluated.

### Complications, adverse events, and additional surgery

We will perform subgroup analyses by testing the same association between our intervention and outcomes within specific subgroups of our sample, namely age, sex, fracture type, duration of surgery, participant center, as well as adverse events and complications. Since these are post-hoc analyses, they should be interpreted with caution. Complications and adverse events will be recorded during the surgical procedure, postoperatively and at follow-up. Intraoperative adverse events include the following: anesthesia, hemorrhage, lesion diameter, and neurological lesion. Postoperative complications include the following: superficial infection, deep infection, neurological lesion, vascular lesion, and hematoma. Adverse effects include the following: death, deep venous thrombosis, pulmonary embolism, pneumonia, urinary infection, acute respiratory insufficiency, multiple organ failure, sepsis, implant breakage or loosening, and reoperation.

All adverse events and complications will be monitored, treated and followed up during the course of the study. Additional surgeries during follow-up that are related to the surgical treatment will be recorded. Additional surgery at the operation level will be considered a complication and a poor result except for patients who require the anterior approach to support a failed anterior column.

### Withdrawal of patients from the study

Patients will be withdrawn from the study if they meet any of the following criteria: (a) the patient chooses to be withdrawn from the study without giving a reason, following the terms in the Helsinki declaration; (b) the patient develops a disease or condition that might interfere with the study results, such as spinal tumor disconnection; or (c) the patient presents any problem related to the study or is non-compliant.

### Data management

The data management of this clinical study will use REDCap™, which is a sophisticated platform that is used to create secure data collection programs and offers an intuitive and comprehensive user interface with a system capable of monitoring and consulting patient records and exporting data for statistical analysis [10]. Although REDCap™ accommodates most clinical research needs, it also offers users the ability to design elaborate and advanced programs [11].

### Statistical considerations

#### Sample size

Considering an expected reduction of two points in the visual analog pain scale (VAS) in 50% of the patients in group B compared to 25% those in group A after surgery with a significance level of 5% and a study power of 80%, and using the Z statistic, where p_1_ (0.5) and p_2_ (0.25) are previously defined, a minimum sample size of 58 patients was obtained, for each of the groups, according to the following formula:

$$ \mathrm{n}={\left( Z\alpha / 2+{Z}_{\beta}\right)}^2\ast \left({p}_1\left( 1-{p}_1\right)+{p}_2\left( 1-{p}_2\right)\right)/{\left({p}_1-{p}_2\right)}^2 $$

Where, Zα/2 is the critical value of the Normal distribution at α/2, i.e., 0.05 and critical value =1.96; Z_β_ is the critical value of the Normal distribution at β, i.e., 0.2 and critical value = 0.84; p_1_ and p_2_ are the previously defined sample proportions of the two groups.

Assuming an alpha of 0.5, a sample size of 58 patients per group was calculated to yield a power of 80% and detect a difference of one point between intervention groups in the 10-point VAS score. Standard differences were estimated to be 1.6 according to previous work (1) and drop-out rates were anticipated to be 28%”.

### Statistical analysis

In the multicenter, randomized clinical trial, comparisons between proportions for the categorical variables will be carried out with the Chi-square test. For verification of differences between measures of central tendency, Student’s t tests for paired samples will be used in the intragroup analysis and for independent samples will be used for the intergroup analysis. In case of comparisons between medians, the Kruskal-Wallis test or another hypothesis test for non-parametric continuous variables will be used.

### Primary outcome

In the analysis of the results regarding the outcomes, we will employ the relative risk (RR) and its 95% confidence interval (95% CI) and derived estimators, such as relative risk reduction (RRR), absolute risk reduction (ARR), risk difference (RD), number needed to treat (NNT), and number needed to damage (NND), emphasizing that in this clinical trial the intention-to-treat (ITT) principle will be used.. To verify the associations among the other variables, univariate analyses followed by multivariate analyses will be performed through logistic regression, also using RR and its 95% CI as association and magnitude estimators.

### Secondary endpoints and safety profile

Considering the secondary outcomes (including minor and major adverse events [AE]), comparing the two groups (A versus B), and for comparing variables between groups A and B, when dealing with proportions, the chi-square test will be used, while for continuous variables the Student’s *t* test (if equal variances) will be used when dealing with averages or the Bonferroni e Benjamini-Hochberg test in the case of medians, in both cases, for independent samples. To minimize the interference of possible confounding variables (in both primary and secondary outcomes), all analyzes will be performed using necessary adjustments such as logistic regression, subgroup analyzes (age, sex, fracture type, duration of surgery, participant center, etc). A significance level of 5% shall be considered in all of the analyses described above.

### Ethical considerations

The study was approved by the Institutional Review Committees (IRCs) of all participating centers that agreed with the proposed study protocol (Trial registration: NCT03316703). Informed consent will be obtained before randomization, and the patients are free to refuse participation. The patients will not receive financial rewards for taking part in the study, and they are free to withdraw from the study at any time without interruption of their treatment and medical care.

## Discussion

Surgical treatment of thoracolumbar fractures traditionally has been performed mainly using an open midline approach involving fixation of the fracture with a pedicle screw-rod system associated with posterolateral bony fusion [4, 5].

Percutaneous pedicle screw fixation (PPSF), which initially was used for the treatment of degenerative spinal diseases, was introduced as an alternative for the treatment of thoracolumbar fractures [6]. PPSF is associated with less intraoperative blood loss, postoperative pain and paravertebral muscles damage [12–15]. However, the radiation exposure and surgery costs are higher [7, 13].

The difference between traditional open and percutaneous minimally invasive surgery for the treatment of thoracolumbar fractures remains controversial, and few randomized trials have been reported in meta-analyses and systematic reviews. Only four randomized studies were reported in the meta-analysis that reported better functional and radiological outcomes of the percutaneous approach than the long-term open approach [9]. Posterolateral bony fusion cannot be performed in PPSF; however, similar clinical and radiological results have been reported between fusion and non-fusion approaches for the posterior fixation of thoracolumbar fractures [16, 17]. The non-fusion concept has been extended to type B1 fractures with good results [13, 18].

The effect of minimally invasive percutaneous pedicle screws is similar to that of traditional open surgery. However, reduced trauma, less bleeding, a shorter operation duration, rapid post-operative recovery, less pain and reduced costs have been reported for PPSF [8, 19]. However, reports concerning the economic costs of minimally invasive surgery for thoracolumbar fractures are controversial because both lower [8] and higher economic costs [7] have been reported for PPSF.

Recently, percutaneous posterior fixation of thoracolumbar fractures has been introduced as an alternative to traditional open posterior fixation. The percutaneous approach presented better results for some clinical and radiological outcomes than the open approach and even reduced the complication rate [20]. However, more high-quality, randomized trials are required to confirm the best option for surgical treatment of thoracolumbar fractures.

## Data Availability

Not applicable in the section.
